# Correlation Between External Body Parameters and Nares-to-Vocal Cord Distance

**DOI:** 10.7759/cureus.101607

**Published:** 2026-01-15

**Authors:** Mohit Jain, Pavan Nayar

**Affiliations:** 1 Department of Anesthesia, Maharishi Markandeshwar Medical College and Hospital, Solan, IND; 2 Department of Critical Care Medicine, Employees' State Insurance Corporation Medical Colleges and Hospitals, Delhi, IND

**Keywords:** blind intubation, external body parameters, fibre optic intubation, nares-to-vocal cord distance, nasopharyngeal airways, nasotracheal intubation

## Abstract

Introduction

Nares-to-vocal cord distance (NVD) is an important anatomical parameter for safe nasotracheal intubation and optimal positioning of nasopharyngeal devices. This study evaluated the relationship between NVD and easily obtained external body measurements in adult surgical patients.

Materials and methods

We conducted a prospective observational study in adults of both sexes undergoing elective surgery under general anesthesia. Using a fiberoptic bronchoscope, we measured NVD from the external nares to the level of the vocal cords. External measurements included height (Ht), weight (Wt), nares-ear tragus distance, nares-mandible angle distance, nares-thyroid distance, thyromental distance, sternomental distance (SMD), sternal length, arm span, and the distance from the external occipital protuberance to the tip of the C7 spinous process (OC7). We assessed correlations between NVD and these anthropometric parameters.

Results

All external body measurements and NVD were greater in men than in women. In the overall cohort and in men, NVD showed the strongest positive correlation with OC7, followed by SMD, Ht, and Wt. In women, NVD also correlated significantly with OC7, Wt, and SMD, although the strength and consistency of these associations were lower than in men. Age and several other external measurements showed little or no meaningful correlation with NVD.

Conclusions

NVD demonstrates clinically relevant associations with simple bedside anthropometric measures, particularly OC7, SMD, Ht, and Wt. These findings suggest that readily obtained external measurements may help clinicians estimate NVD when planning nasotracheal intubation or positioning nasopharyngeal devices, especially in settings where fiberoptic assessment is unavailable. Further research across broader, more diverse populations is needed to refine these relationships and support the development of practical prediction tools for airway management.

## Introduction

Nasotracheal intubation is used to secure the airway and induce anesthesia for head and neck surgeries, allowing better isolation and good surgical access for intraoral or facial procedures [1]. Nasotracheal intubation has the advantage of causing less neck movement and does not require mouth opening. It can be performed blindly or with a fiberoptic technique. Fiberoptic intubation is the gold-standard technique for anticipated difficult intubation, whereas in resource-poor regions and in emergencies, blind nasotracheal intubation is more common [2].

Nasotracheal intubation can be performed in the sitting position, which is advantageous in patients who cannot tolerate the supine position, such as those with congestive heart failure. Other advantages of a nasotracheal tube include the absence of tube biting or tongue manipulation and a less pronounced gag reflex compared to oral intubation. A nasotracheal tube also interferes less with the management of oral injuries, and its stabilization and care are easier than for an orotracheal tube [3]. Patients generally tolerate nasotracheal tubes better, with easier movement in bed, and this may be associated with less reflex salivation than with oral tubes.

The distance from the nares to the vocal cords can guide the correct placement of the endotracheal tube, nasopharyngeal airway, and temperature sensor. Clinicians can measure the nares-to-vocal cord distance (NVD) with a fiberoptic bronchoscope (FOB), which can help position the endotracheal tube during blind nasal intubation [1] and select the correct size of nasopharyngeal airway [4]. This study was conducted to correlate NVD with various external body parameters and to assess the relationship between a novel parameter (i.e., the distance from the external occipital protuberance to the tip of the C7 spinous process (OC7)) and NVD. Particular focus was placed on OC7 due to its anatomical proximity and pilot data suggesting the strongest predictive performance.

## Materials and methods

We conducted this prospective study in the Department of Anaesthesia and Intensive Care, V.M.M.C. and Safdarjung Hospital, New Delhi, from October 2016 to March 2019, enrolling 50 male and 50 female patients with American Society of Anesthesiologists (ASA) physical status I or II, aged 18-60 years, who were admitted to the hospital and scheduled to undergo elective surgery.

We calculated a priori sample size using Fisher's z-transformation formula for correlation studies:

$N = \left[ \frac{Z_{\alpha/2} + Z_{\beta}}{C(r)} \right]^2 + 3$

where Zα/2 is 1.96 (α = 0.05, two-sided test), Zβ is 1.28 (90% power), and C(r) is $\tfrac{1}{2} \ln \left( \frac{1+r}{1-r} \right)$ (Fisher's z-transformation).

Minimum sample size was 44 males + 38 females = 82 total. We enrolled n = 100 (50 males, 50 females) to further reduce the margin of error.

The Institutional Ethics Committee of Vardhman Mahavir Medical College and Safdarjung Hospital, New Delhi, approved the study (approval number: IEC/VMMC/SJH/Thesis/October/2016/7). The study was registered with CTRI under reference no. CTRI/2018/05/014302. We informed all patients about the nature of the study and obtained valid written informed consent.

Inclusion criteria were adults (18-60 years) of both sexes and of ASA I and II physical status who were undergoing elective surgery under general anesthesia at our institution. Exclusion criteria were patient refusal; body mass index >35 kg/m²; history of obstructive sleep apnea; congenital malformation of the face, neck, upper airway, spine, or thoracic cage; prior surgical interventions in these regions; nasal pathology or trauma; anticipated difficult intubation or difficult airway; reactive airway disease; need for rapid sequence induction (including obstetric patients); and abnormal coagulation profile. All patients underwent a thorough pre-anesthetic evaluation, including a detailed medical history, general physical and systemic examination with airway assessment, and relevant laboratory investigations. We counseled all patients for fiberoptic examination in the pre-anesthetic clinic.

We recorded demographic variables (age and sex) and obtained the following physical measurements (primary focused on OC7 as lead predictor) in centimeters using a measuring tape in standing position: height (Ht), weight (Wt), the nares-ear tragus distance (NED; from the lateral border of the external nares to the ear tragus), nares-mandible angle distance (NMD; from the lateral border of the external nares to the mandibular angle), and nares-thyroid distance (NTD), which we defined as the sum of the nares-mandibular angle distance and the mandibular angle-thyroid cartilage (tubercle) distance. We measured the thyromental distance (TMD; from the thyroid cartilage tubercle to the chin in full neck extension), sternomental distance (SMD; from the suprasternal notch to the chin in full neck extension), and sternal length (SL; from the suprasternal notch to the junction of the body of the sternum with the xiphoid process). We recorded arm span (AS; from one fingertip to the other with the arms outstretched horizontally at shoulder Ht at a 180° angle) and the distance from the external occipital protuberance to the tip of the spinous process of the C7 vertebra in full neck flexion (chin touching the chest), designated as OC7. External parameters were measured by trained assistants who were blinded to NVD status. Inter-rater reliability for OC7 (n = 20) was excellent, with an intraclass correlation coefficient of 0.92.

All patients fasted from midnight before surgery and received oral premedication with ranitidine 150 mg, metoclopramide 10 mg, and alprazolam 0.25 mg at 10 pm the night before surgery and again at 6 am on the morning of surgery with one to two sips of water. In the operating theater, after confirming patient identity and details, we attached a multiparameter patient monitor. We recorded the following baseline parameters and monitored them continuously during the perioperative period: heart rate, noninvasive blood pressure, peripheral oxygen saturation, and respiratory rate. We also monitored the electrocardiogram and end-tidal carbon dioxide. We prepared all anesthetic drugs freshly and checked the anesthesia workstation. We secured intravenous (IV) access with an 18-gauge cannula and initiated an infusion of Ringer’s lactate solution.

Ten minutes before induction of general anesthesia, we instilled two drops of xylometazoline hydrochloride into each nostril. The patient then sniffed 2% lignocaine jelly into the more patent nostril. We administered IV midazolam 1-2 mg, followed by IV fentanyl citrate 1.5-2 mcg/kg given slowly over 30 seconds. We induced anesthesia with IV propofol 2 mg/kg until loss of verbal response. We confirmed the ability to ventilate the lungs with 100% oxygen (O₂) and then administered IV vecuronium bromide 0.1 mg/kg as a muscle relaxant. We provided intermittent positive-pressure ventilation with 100% O₂ and 0.5-1.0% isoflurane for three minutes.

**Figure 1:**
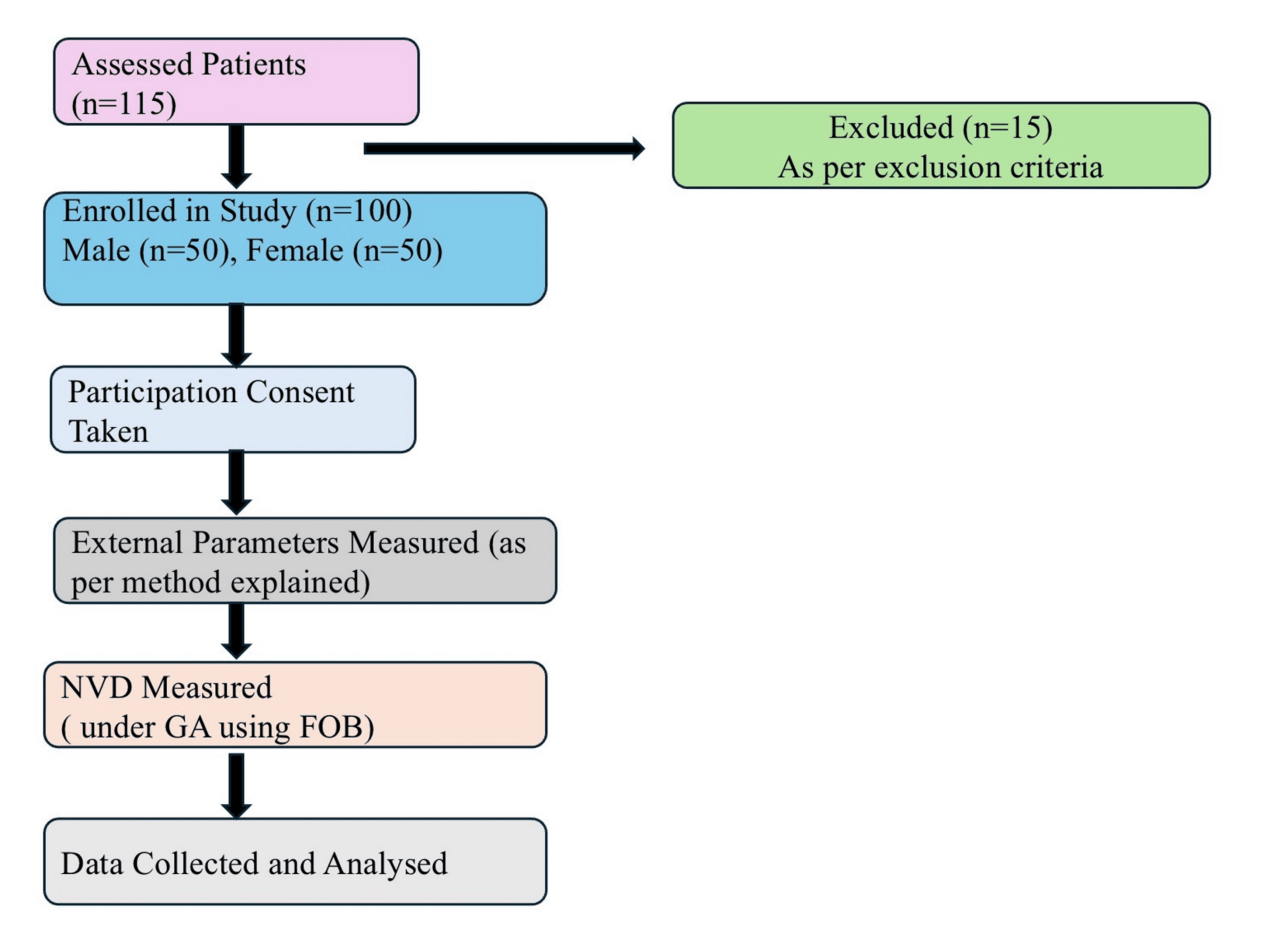
CONSORT style study flow diagram From 115 patients assessed for eligibility, 15 were excluded per criteria, leaving 100 enrolled (50 males, 50 females). External body parameters were measured, followed by NVD measurement using FOB under standardized general anesthesia protocol, with subsequent statistical analysis. CONSORT: Consolidated Standards of Reporting Trials, NVD: nares-to-vocal cord distance, FOB: fiberoptic bronchoscope

After adequate muscle relaxation, we lubricated a FOB (Karl Storz GmbH, outer diameter 5.2 mm) and inserted it into the nostril assessed as more patent. We advanced the FOB until the tip reached the level of the vocal cords, with the head in the neutral position. During the procedure, we administered continuous oxygen insufflation at 4 L/min via the FOB’s working channel. A single attending consultant performed all fiberoptic nasal endoscopic procedures, ensuring intra-rater consistency in the supine, neutral head position, and we continuously monitored vital parameters. We used tape to mark the length of the FOB at the nares when the tip was precisely positioned between the vocal cords. We then withdrew the FOB and intubated the patient orally using an appropriately sized cuffed orotracheal tube by direct laryngoscopy, recording the time of intubation. Anesthesia was then maintained as required for surgery. We measured the distance from the FOB tip to the tape mark in centimeters, which corresponded to the NVD. The complete study methodology is illustrated in Figure 1.

Statistical analysis

We presented categorical variables as counts and percentages and continuous variables as means ± standard deviations and medians. We tested data normality with the Kolmogorov-Smirnov test and used nonparametric tests when the data were not normally distributed. We compared quantitative variables between age groups using the Kruskal-Wallis test. We used the Spearman rank correlation coefficient to assess the association of various parameters with NVD. We considered a p-value of <0.05 statistically significant. We entered the data into a Microsoft Excel spreadsheet (Microsoft Corp., Redmond, WA, USA) and performed statistical analysis using SPSS Statistics version 21.0 (IBM Corp. Released 2012. IBM SPSS Statistics for Windows, version 21.0. Armonk, NY: IBM Corp.). Thirty Spearman correlation analyses were performed, with primary emphasis on the strongest associations (OC7: r = 0.644, p < 0.0001). After Bonferroni correction, the adjusted significance level (α = 0.0017) confirmed the statistical significance of the strongest correlates.

## Results

**Figure 2:**
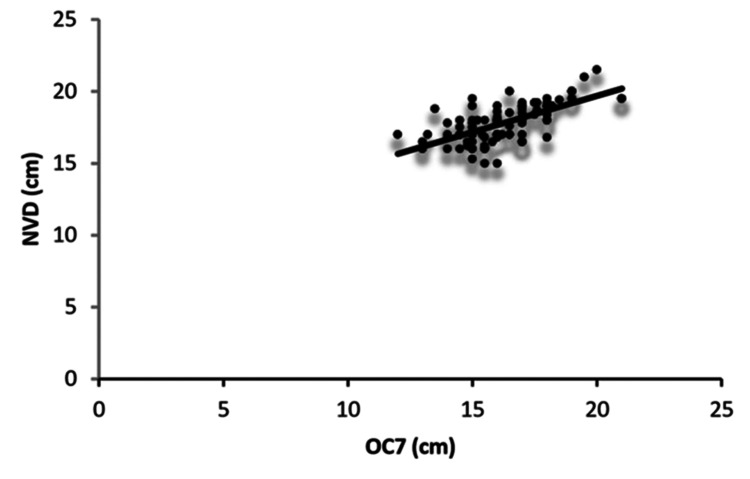
Correlation between OC7 distance and NVD in all participants Scatterplot showing the correlation between OC7 and NVD in men and women combined. NVD: nares-to-vocal cord distance, OC7: distance from the external occipital protuberance to the tip of the C7 spinous process in full neck flexion, chin touching the chest

All external body measurements, including Ht, Wt, NED, NMD, NTD, TMD, SMD, SL, AS, OC7, and NVD, had higher values in men than in women. Descriptive statistics for the overall cohort and stratified by sex are presented in Tables 1-3. In the combined sample of men and women, the strength of correlation between NVD and external measurements, in decreasing order, was OC7 > Ht > AS > SMD > SL > Wt > TMD > NTD > NMD > NED (Table 4, Figure 2). There was no correlation between NVD and age (r = -0.102, p = 0.3115), and all other correlations were statistically highly significant.

**Table 1:** External body parameters and NVD in men and women combined (N = 100) AS: arm span, NED: nares-ear tragus distance, NMD: nares-mandible angle distance, NTD: nares-thyroid distance, NVD: nares-to-vocal cord distance, OC7: distance from the external occipital protuberance to the tip of the C7 spinous process in full neck flexion, SD: standard deviation, SL: sternal length, SMD: sternomental distance, TMD: thyromental distance

Parameter	Mean ± SD	Median	Min-max	Interquartile range
Age (years)	36.85 ± 10.44	35	20-60	28.500-45
Height (cm)	160.45 ± 7.79	160	141-176	156-166
Weight (kg)	58.65 ± 10.11	60	35-80	51-65
NED (cm)	11.86 ± 1.11	11.5	10-15	11-12.600
NMD (cm)	10.89 ± 1.18	10.7	9-14	10-11.500
NTD (cm)	18.33 ± 1.49	18	14.5-23	17-19.500
TMD (cm)	8.09 ± 1.12	8	6-11	7-9
SMD (cm)	17.30 ± 1.68	17	13-20.5	16-18.800
SL (cm)	16.72 ± 1.62	17	13.5-22	15.650-17.500
AS (cm)	164.39 ± 8.86	164.5	136-183	158.950-171
OC7 (cm)	16.25 ± 1.71	16	12-21	15-17.250
NVD (cm)	17.80 ± 1.30	17.8	15-21.5	17-19

**Table 2:** External body parameters and NVD in men (N = 50) AS: arm span, NED: nares-ear tragus distance, NMD: nares-mandible angle distance, NTD: nares-thyroid distance, NVD: nares-to-vocal cord distance, OC7: distance from the external occipital protuberance to the tip of the C7 spinous process in full neck flexion, SD: standard deviation, SL: sternal length, SMD: sternomental distance, TMD: thyromental distance

Parameter	Mean ± SD	Median	Min-max	Interquartile range
Age (years)	38.04 ± 12.14	35.5	20-60	28-48
Height (cm)	166.06 ± 5.53	166	155-176	162-170
Weight (kg)	63.10 ± 9.32	64.5	42-80	56-70
NED (cm)	11.93 ± 0.99	11.55	10-14.5	11.200-13
NMD (cm)	11.04 ± 1.11	11	9-14	10.500-12
NTD (cm)	18.83 ± 1.19	19	16.5-21.4	18-19.500
TMD (cm)	8.66 ± 0.78	9	6.5-10	8-9
SMD (cm)	18.10 ± 1.55	18.5	14.5-20.5	17-19.500
SL (cm)	17.54 ± 1.52	17	14-22	17-18
AS (cm)	169.88 ± 6.32	170	158.9-183	165-174
OC7 (cm)	17.03 ± 1.72	17	13.5-21	16-18
NVD (cm)	18.59 ± 1.09	18.75	16.5-21.5	17.800-19.200

**Table 3:** External body parameters and NVD in women (N = 50) AS: arm span, NED: nares-ear tragus distance, NMD: nares-mandible angle distance, NTD: nares-thyroid distance, NVD: nares-to-vocal cord distance, OC7: distance from the external occipital protuberance to the tip of the C7 spinous process in full neck flexion, SD: standard deviation, SL: sternal length, SMD: sternomental distance, TMD: thyromental distance

Parameter	Mean ± SD	Median	Min-max	Interquartile range
Age (years)	35.66 ± 8.36	34	24-55	30-40
Height (cm)	154.84 ± 5.26	156	141-163	150-159
Weight (kg)	54.20 ± 8.90	55.5	35-74	48-61
NED (cm)	11.79 ± 1.23	11.5	10-15	11-12.500
NMD (cm)	10.73 ± 1.24	10.5	9-14	10-11
NTD (cm)	17.82 ± 1.60	17.5	14.5-23	17-19
TMD (cm)	7.53 ± 1.13	7.2	6-11	6.500-8.500
SMD (cm)	16.51 ± 1.42	16.2	13-19.5	15.500-17
SL (cm)	15.89 ± 1.27	16	13.5-18.6	15-17
AS (cm)	158.90 ± 7.56	160	136-176	154-163
OC7 (cm)	15.48 ± 1.31	15.5	12-18	15-16
NVD (cm)	17.02 ± 0.99	17	15-19.5	16.500-17.800

**Table 4:** Correlation of NVD with external body parameters AS: arm span, NED: nares-ear tragus distance, NMD: nares-mandible angle distance, NTD: nares-thyroid distance, NVD: nares-to-vocal cord distance, OC7: distance from the external occipital protuberance to the tip of the C7 spinous process in full neck flexion, SL: sternal length, SMD: sternomental distance, TMD: thyromental distance

Parameter	NVD (men and women, N = 100)		NVD (men, N = 50)		NVD (women, N = 50)	
	Correlation coefficient	p-value	Correlation coefficient	p-value	Correlation coefficient	p-value
Age (years)	-0.102	0.3115	-0.344	0.014	0.007	0.963
AS (cm)	0.548	<0.0001	0.380	0.006	0.032	0.826
Height (cm)	0.584	<0.0001	0.460	0.001	-0.016	0.910
NED (cm)	0.225	0.0244	0.333	0.018	0.068	0.637
NMD (cm)	0.246	0.0135	0.294	0.038	0.131	0.365
NTD (cm)	0.310	0.0017	0.331	0.019	-0.034	0.812
OC7 (cm)	0.644	<0.0001	0.807	<0.0001	0.306	0.030
SL (cm)	0.525	<0.0001	0.289	0.042	0.087	0.549
SMD (cm)	0.537	<0.0001	0.478	0.0004	0.297	0.036
TMD (cm)	0.363	0.0002	0.046	0.749	0.106	0.464
Weight (kg)	0.506	<0.0001	0.434	0.002	0.301	0.034

Ht and Wt were higher in men than in women, whereas age was similar between the groups. The mean values of NVD, NED, NMD, NTD, TMD, SMD, SL, AS, and OC7 were also higher in men than in women. In men, the correlations between NVD and OC7, SMD, Ht, and Wt were highly significant (all p < 0.001). The correlation coefficient (r) for NVD with OC7 was 0.807, with SMD was 0.478, with Ht was 0.460, and with Wt was 0.434. Correlations between NVD and AS, NED, NTD, NMD, and SL were also statistically significant, with correlation coefficients of 0.380, 0.333, 0.331, 0.294, and 0.289, respectively. There was very poor correlation between NVD and age (r = -0.344, p = 0.014) and between NVD and TMD (r = 0.046, p = 0.749). In women, NVD correlated significantly with OC7, Wt, and SMD, with correlation coefficients of 0.306, 0.297, and 0.301, respectively. Correlations of NVD with NMD, TMD, SL, NED, AS, age, Ht, and NTD were not significant.

**Figure 3:**
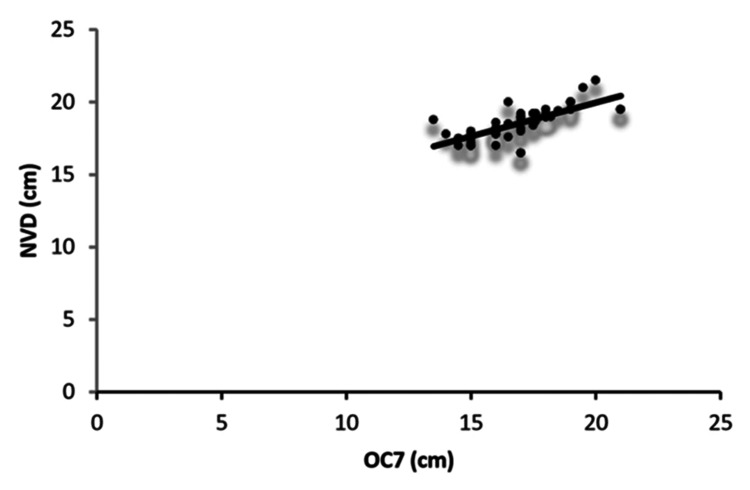
Correlation between OC7 distance and NVD in men Scatterplot showing the correlation between OC7 and NVD in men. NVD: nares-to-vocal cord distance, OC7: distance from the external occipital protuberance to the tip of the C7 spinous process in full neck flexion, chin touching the chest

In men, the correlation of NVD with body measurements in decreasing order was OC7 > SMD > Ht > Wt > AS > NED > NTD > NMD > SL > TMD (Figure 3). NVD showed very poor correlation with age and TMD, and all correlations were statistically significant except for the association between NVD and TMD (p = 0.749, > 0.05).

**Figure 4:**
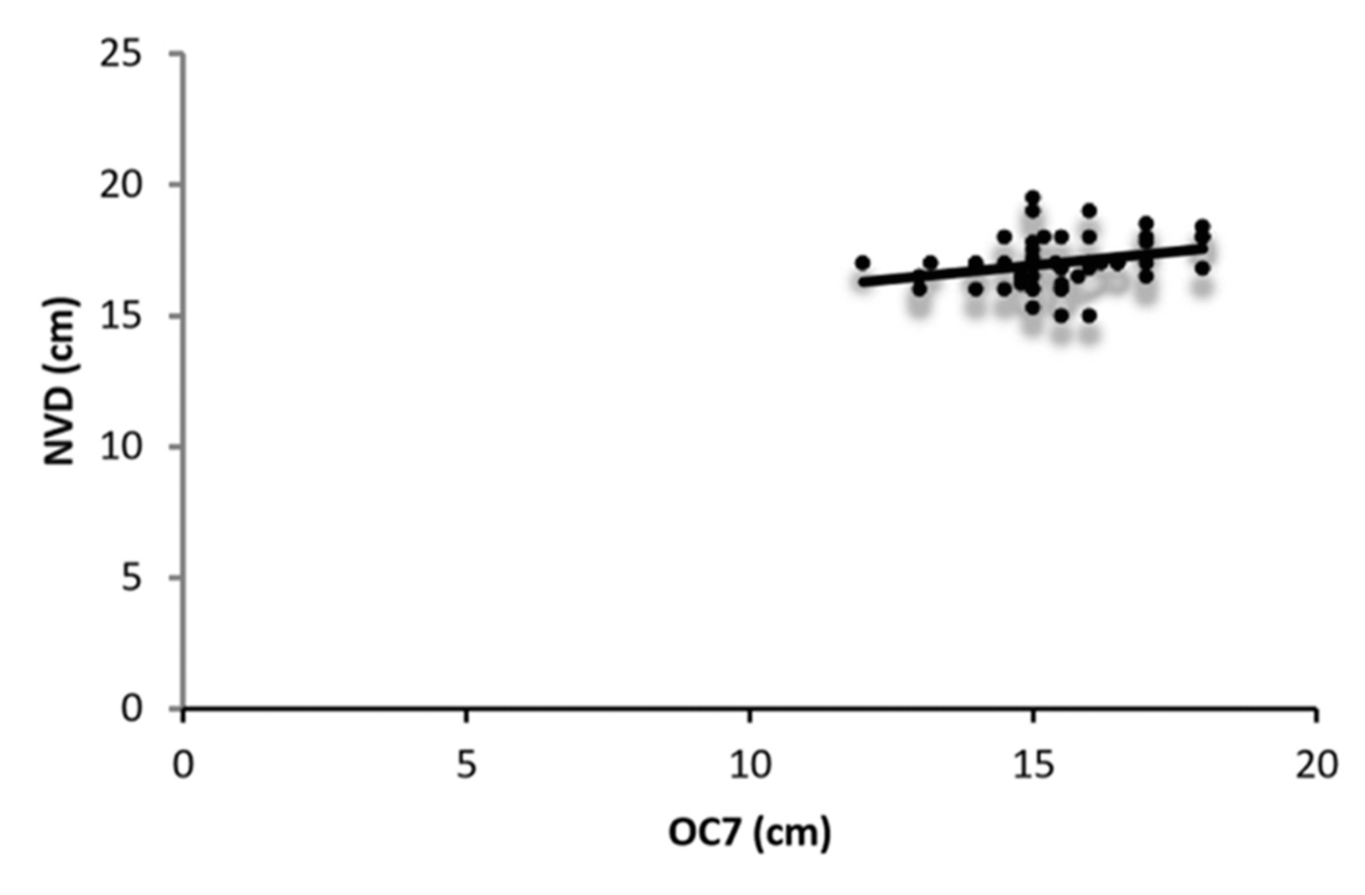
Correlation between OC7 distance and NVD in women Scatterplot showing the correlation between OC7 and NVD in women. NVD: nares-to-vocal cord distance, OC7: distance from the external occipital protuberance to the tip of the C7 spinous process in full neck flexion, chin touching the chest

In women, the correlation of NVD with external body measurements, in decreasing order, was OC7 > Wt > SMD > NMD > TMD > SL > NED > AS > age > Ht > NTD (Figure 4). Correlations between NVD and OC7, Wt, and SMD were statistically significant, whereas those with the other parameters were not.

## Discussion

Several studies have evaluated associations between external body measurements, such as nares-epiglottis (N-E) distance [4] and NVD [5], similar to the approach used in the present study. We also assessed a novel parameter, OC7, which has not been evaluated in prior studies, and demonstrated a statistically significant association with NVD in both men and women. These findings suggest that OC7 may be a useful addition to bedside airway assessment and warrant further investigation to confirm its reproducibility and clinical utility in guiding airway management.

OC7 showed the strongest NVD correlation (r = 0.644 overall, r = 0.807 males, r = 0.463 females), validating it as the primary predictor. Clinical benefits include accurate ETT depth positioning, which prevents vocal cord impingement (2 cm above) and endobronchial intubation, and optimal ETT size selection, which reduces nasal trauma and ensures airway patency. Additionally, it allows for safer blind nasal intubation, which is critical for elective head, neck, and maxillofacial surgeries when an FOB is unavailable. Overall, this approach reduces airway complications and enables standardized NVD prediction across typical surgical patients (ASA I-II).

The present study showed that Ht correlated strongly with NVD in men, consistent with Stoneham’s findings [4]. This suggests that it may be more accurate and practical to estimate nasopharyngeal airway length using body Ht, as Ht is easier to obtain than N-E distance and can be used more routinely in clinical practice as an independent variable.

Stoneham evaluated the correlation between the N-E distance and the distance from the tip of the nose to either the mandibular angle or the tragus of the ear, but did not observe any significant correlations [4]. However, the authors clearly identified a relationship between N-E distance and subject Ht in that study. Estimation of NVD is helpful for optimal visualization of the vocal cords during fiberoptic intubation. It is also useful for blind nasal intubation, positioning of a nasal airway or an endotracheal tube used as a nasopharyngeal airway, and placement of a temperature sensor [4,6].

In a study by Han et al., the NVD was 18.3 ± 0.8 cm in men and 16.3 ± 0.7 cm in women [7]. The relationship between the NVD and body Ht (p < 0.001, r = 0.755) and between NVD and the NED (p < 0.001, r = 0.636) showed a significant correlation, but the nares-to-angle-of-mandible distance did not (p = 0.075) [7]. In the present study, the NVD was 18.59 ± 1.09 cm in men and 17.02 ± 0.99 cm in women. In men, the correlation between NVD and Ht was statistically significant (p = 0.001, r = 0.46), whereas in women, it was not (p = 0.910, r = -0.016).

Eagle et al. conducted a study examining the relationship between a person’s Ht and upper airway dimensions in adult subjects [8]. They derived a formula for nasotracheal tube length (external nares to the midpoint of the trachea): tube length = subject Ht (cm) / 10 + 8. In that study, the mean length from nose to vocal cords was 18 cm in women and 19.8 cm in men. The correlation between Ht and the distance from the vocal cords to the teeth was good (r = 0.53), and the correlation between Ht and the distance from the external nares to the vocal cords was even stronger (r = 0.69) [8]. In the present study, we likewise found a strong correlation between Ht and NVD in men (r = 0.46).

Kim et al. studied 211 children aged one to 10 years undergoing elective surgery to measure the N-E distance and NVD and to develop an algorithm to predict these distances using anatomical landmarks and pediatric data [9]. They measured the N-E distance using a nasogastric tube after induction of general anesthesia and measured the NED and nares-to-mandible distances with a measuring tape. They found significant correlations between N-E distance and NVD distance with age, Wt, Ht, and external measurements. They concluded that the N-E distance and NVD can be predicted from Ht and the nares-to-mandible distance in young children [9]. Although the study by Kim et al. was conducted in a pediatric population, the pattern of correlations is similar to that observed in our study.

Sareen et al. studied 100 Indian subjects (50 male and 50 female) and found that the mean NVD was 18.5 cm in men and 15.87 cm in women [10]. They measured the distance between the external nares and the vocal cords and analyzed its correlation with external body parameters, including Ht, NED, NMD, NTD, TMD, SMD, SL, and AS. They reported significant correlations of NVD with Ht, SL, and AS in both men and women. In men, the correlation coefficients (r) for NVD with SL, AS, and Ht were 0.759, 0.561, and 0.463, respectively, whereas in women, the correlation coefficients for NVD with SL, Ht, and AS were 0.801, 0.555, and 0.499, respectively [10]. The findings of the present study are broadly similar to those reported by Sareen et al. [10].

Limitations

This study has several limitations that should be considered when interpreting the findings. First, it was conducted at a single tertiary care center with a relatively small, homogeneous sample of adults with ASA physical status I or II undergoing elective surgery, which may limit generalizability to other settings, age groups, emergency cases, or patients with significant comorbidities. The exclusion of individuals with obesity, obstructive sleep apnea, airway pathology, prior airway surgery, or anticipated difficult airway further restricts the applicability of these results to higher-risk populations in whom accurate estimation of NVD may be most challenging. Second, all measurements of external body parameters and NVD were obtained using simple bedside tools and a single technique under general anesthesia, without formal assessment of interobserver or intraobserver variability, so measurement error and operator dependence cannot be excluded. Third, the observational design focused on correlation analysis and did not derive or prospectively validate a predictive formula, nor did it link anatomical estimates to clinical outcomes such as ease of intubation, number of attempts, or airway-related complications. Finally, the study population consisted of Indian adults, and craniofacial or anthropometric differences across ethnic groups may limit the direct applicability of these correlations to other populations, underscoring the need for external validation in diverse cohorts.

## Conclusions

This study examined how NVD relates to external body measurements in adult men and women undergoing elective surgery. We found that NVD was greater in men than in women and that it correlated most consistently with the distance from the external occipital protuberance to the C7 vertebra, SMD, Ht, and Wt. In women, the association between NVD and these external measurements was present but generally weaker and more limited to a smaller set of parameters. These findings suggest that simple bedside measurements, particularly those reflecting overall body size and upper spinal length, can help clinicians estimate NVD when planning nasotracheal intubation or positioning other nasopharyngeal devices. Using such anatomical predictors may improve the accuracy of tube placement, reduce the need for repeated attempts, and enhance airway safety in settings where fiberoptic assessment is unavailable or impractical. Further studies in broader populations and clinical contexts are needed to refine these relationships and support the development of easy-to-use prediction tools and formulas for routine airway management.
